# Neuraminidase (NA) 370-Loop Mutations of the 2009 Pandemic H1N1 Viruses Affect NA Enzyme Activity, Hemagglutination Titer, Mouse Virulence, and Inactivated-Virus Immunogenicity

**DOI:** 10.3390/v14061304

**Published:** 2022-06-14

**Authors:** Ting-Hsuan Chen, Chung-Chu Chen, Suh-Chin Wu

**Affiliations:** 1 Institute of Biotechnology, National Tsing Hua University, Hsinchu 30013, Taiwan; sadam1114@gmail.com; 2 Department of Internal Medicine, MacKay Memorial Hospital, Hsinchu 30071, Taiwan; 4059@mmh.org.tw; 3 Teaching Center of Natural Science, Minghsin University of Science and Technology, Hsinchu 30401, Taiwan; 4 Department of Medical Science, National Tsing Hua University, Hsinchu 30013, Taiwan; 5 Adimmune Corporation, Taichung 42723, Taiwan

**Keywords:** influenza virus, neuraminidase, 370-loop, vaccine

## Abstract

Hemagglutinin (HA) and neuraminidase (NA) are the two major envelope proteins of influenza viruses. The spatial organization of HA and NA on the virus surface needs to be optimized to promote viral fitness, host specificity, transmissibility, infectivity, and virulence. We previously demonstrated that the recombinant NA protein of the 2009 pandemic H1N1 (pH1N1) with the I365T/S366N mutation in the NA 370-loop elicited higher NA-inhibition antibody titers against the homologous pH1N1 virus and three heterologous H5N1, H3N2, and H7N9 viruses in mice. In this study, we used PR8-based reverse genetics (RG) by replacing the HA and NA genes of A/Texas/05/2009 pH1N1 virus to obtain the wild-type pH1N1 and three NA 370-loop mutant viruses of pH1N1 (I365T/S366N), RG pH1N1 (I365E/S366D), and RG pH1N1 (I365T/S366A). Our results revealed that the viral NA enzyme activity increased for the RG pH1N1(I365T/S366N) and RG pH1N1 (I365E/S366D) viruses but reduced for the RG pH1N1 (I365T/S366A) virus. The increased or decreased NA enzyme activity was found to correlate with the increase or decrease in HA titers of these NA 370-loop mutant viruses. All of these three NA 370-loop mutant RG pH1N1 viruses were less virulent than the wild-type RG pH1N1 virus in mice. Immunizations with the inactivated viruses carrying the three NA 370-loop mutations and the wild-type RG pH1N1 virus were found to elicit approximately the same titers of NA-inhibition antibodies against H1N1 and H5N1 viruses. These results may provide information for developing NA-based influenza virus vaccines.

## 1. Introduction

Influenza viruses belong to the *Orthomyxoviridae* family and consist of single-stranded eight-segment negative-sense genomic RNA, helical viral ribonucleoprotein (RNP) complexes (RNA segments NP, PB2, PB1, and PA), and four viral envelope proteins (hemagglutinin (HA), neuraminidase (NA), M1 matrix protein, and M2 ion channel protein) [1]. HA and NA are the two major surface envelope proteins of influenza viruses. The antigenic diversity of HA and NA are used to determine the influenza subtype. Moreover, both HA and NA recognize sialic acid (SIA) in host cells [2]. HA is a receptor-binding glycoprotein that binds to SIA to initiate viral infection and is the main antigen eliciting neutralizing antibodies and protection [3]. NA is a receptor-destroying enzyme that cleaves the SIA linkage between HA and the sialylated receptors of host cells to facilitate offspring virus release [4]. NA can also remove SIA decoy receptors from mucins, cilia, and cellular glycocalyx in respiratory airways to help viruses penetrate the heavily sialylated mucus layer overlaying on host cells to promote virus entry [5,6,7]. The spatial organization of HA and NA on the virus surface was shown to be correlated with the direction of virus movement in the host mucus layer [8]. Moreover, NA can interact with HA to facilitate viral movement on the cell surface for virus migration [7,9]. HA and NA are functional antagonists of each other, and the balance between HA binding affinity and NA enzyme activity needs to be optimized to promote viral fitness, host specificity, transmissibility, infectivity, and virulence [2,10].

NA was shown to elicit NA-inhibition antibodies via natural infection or vaccination in humans [11], as well as animal models of mice [12], ferrets [13], rabbits [14], swans and ibises [15], and non-human primates [16]. NA-inhibition antibody titers are inversely correlated with illness severity, symptoms, and disease duration [17]. Moreover, several studies have reported that NA-inhibition antibodies inhibit the NA activity of heterologous viruses carrying the same NA subtype [18]. NA-inhibition antibodies induced by seasonal trivalent influenza vaccines were also shown to provide protection against H5N1 challenges in ferrets [13]. In addition, cross-reactive NA-inhibition antibodies against H5N1 viruses were reported in ferrets with live attenuated vaccine immunization [19].

We previously demonstrated that recombinant N1 protein with the I365T/S366N mutation in the NA 370-loop elicited more potent cross-reactive NA-inhibition antibody titers against pH1N1, H5N1, H3N2, and H7N9 viruses [12]. In this study, we used the PR8-based reverse genetics (RG) system [20] to obtain engineered RG pH1N1 virus and three mutant viruses of RG pH1N1(I365T/S366N), RG pH1N1(I365T/S366A), and RG pH1N1(I365E/S366D). We investigated the viral NA enzymatic activity, viral HA titer, mouse virulence of these mutant RG viruses, and the hemagglutination inhibition (HI) and NA-inhibition antibody titers in sera elicited by immunization with these formalin-inactivated RG viruses. These results may provide useful information for NA-based influenza vaccine development.

## 2. Materials and Methods

### 2.1. Generation of RG Viruses

Eight plasmids containing the cDNA sequences of PB2, PB1, PA, NP, M, and NS from A/PR/8/1934(H1N1), as well as those of HA and NA of pH1N1 (A/Texas/05/2009), were cloned into a modified pcDNA3.1 plasmid containing an RNA polymerase II promoter (CMV promoter) and a human RNA polymerase I promoter (PolIp) similar to the generation of pHW2000 [20]. Plasmids carrying mutant N1 genes (I365T/S366N mutant N1, I365E/S366D mutant N1, and I365T/366A mutant N1) were obtained by site-directed PCR. The eight plasmids (1 µg/plasmid) were incubated with 32 µL of TransIT^®^-LT1 transfection reagent (Mirus Bio, Madison, WI, USA) in 800 µL of OPTI-MEM at room temperature for 45 min. Plasmid transfection reagent mixtures were added to a co-culture of MDCK/293T (4 × 10^5^/4.5 × 10^5^) in a 6-well plate. Twenty-four hours after transfection, the medium was changed to 2.5 mL of fresh OPTI-MEM containing 0.5 µg/mL TPCK-trypsin. After 72 h of incubation at 37 °C, the supernatant was collected and tested by hemagglutination assay to confirm virus rescue. The viruses were further amplified in MDCK cells. Virus titers were measured using plaque assays. For virus growth curve determination, 2 × 10^7^ MDCK cells prepared in T175 flasks were infected with viruses (MOI = 0.001) in 20 mL of MEM-α with 0.5 µg/mL TPCK-trypsin. After incubation for 1 h for virus absorption, the cells were washed with PBS and incubated in fresh MEM-α containing 0.5 µg/mL TPCK-trypsin. The viral titers of samples collected every 12 h for 72 h were measured by plaque assays and plotted as growth curves.

### 2.2. Influenza Virus Plaque Assay

MDCK cells (9.5 × 10^5^) in 2 mL of DMEM were seeded in a 6-well plate and incubated at 37 °C for 48 h. After washing the cells twice with 1 mL of PBS, 1 mL of two-fold serially diluted virus in MEM-α with 0.5 µg/mL TPCK-trypsin was added to the cells. After an hour of incubation at 37 °C for virus absorption, the supernatant was removed from each well, and the cells were washed once with 1 mL of PBS. The cells were then covered with 3 mL of overlay gel (0.5% low melting agarose in MEM-α with 0.5 µg/mL TPCK-trypsin) and incubated at 37 °C for 48 h for plaque formation. Then, 1 mL of 4% formalin (Sigma, Burlington, MA, USA) was added to the cells for over 6 h. After removing the overlay gel, the plaques were stained with 1% crystal violet and washed with tap water.

### 2.3. NA Enzyme Activity by MUNANA Assay and Kinetic Measurement

An NA-Fluor™ influenza neuraminidase assay kit (Thermo Fisher Scientific, Waltham, MA, USA) was used to perform the MUNANA (’-(4-methylymbelliferyl)-α-d-N-acetylneuraminic acid) assays. Briefly, 50 µL of two-fold serially diluted viruses (starting from 10^6^ PFU) in 1× assay buffer were co-incubated with an equal volume of 200 µM MUNANA substrate at 37 °C for 60 min in a black 96-well plate in the dark. Wells without viruses were used as background controls. Then, the virus-substrate mixtures were treated with 100 µL of NA-Fluor stop solution to stop the reaction. Finally, the plate was read using a VICTOR3 Multilabel plate reader (Wallac, TURKU, Varsinais-Suomi, Finland). The excitation and emission wavelengths were 355 and 460 nm, respectively. The values of relative fluorescence units (RFU) from each sample minus the value from the background control were plotted as curves. The procedures of the MUNANA kinetic assay were performed as described in a previous report with minor modifications [21]. Briefly, 50 µL of 10^6^ PFU of viruses was added to 50 µL of two-fold serially diluted MUNANA substrate (from 2000 µM to 0 µM) and incubated at 37 °C in a black 96-well plate in the dark. The fluorescence values released by cleaved MUNANA were measured using a VICTOR3 Multilabel Plate Reader every 60 s for 60 min. The excitation and emission wavelengths were 355 and 460 nm, respectively. The RFU values recorded at each time point for different MUNANA concentrations were plotted as curves. The catalytic velocity of each reaction with different MUNANA concentrations within 3 min was calculated and plotted as a Michaelis–Menten plot. Then, the Michaelis–Menten plot was transformed into a Lineweaver–Burk curve by transforming the X-axis from MUNANA into 1/(MUNANA) and the Y-axis from V to 1/V. The maximum velocity of NA activity (Vmax) and the Km of NA were calculated by nonlinear regression using GraphPad Prism version 6 software (La Jolla, CA, USA).

### 2.4. NA Enzyme Activity by Enzyme-Linked Lectin Assay (ELLA)

The NA activity of the viruses on multivalent fetuin substrates was determined using ELLA as previously described with minor modifications [12,22,23]. Briefly, ELISA plates coated with 50 μg/mL fetuin ((Sigma, Burlington, MA, USA) were washed with PBST (PBS with 0.05% Tween-20) and blocked with blocking buffer (1% BSA in PBS) for 2 h. Two-fold serially diluted viruses (from 10^6^ PFU) in 100 μL of blocking buffer were added to the plate and incubated at 37 °C for 1 h for SIA cleavage. After three washes with PBST, the desialylated O-linked glycans and desialylated N-linked glycans were probed with 100 μL of 2.5 μg/mL biotin-conjugated peanut agglutinin (PNA) (Vector Laboratories, Burlingame, CA, USA) and 100 μL of 1.25 μg/mL biotinylated lectin from erythrina cristagalli (ECA) (Vector Laboratories), respectively, in separate sets of experiments at RT for 1 h. After washing three times with PBST, the binding of PNA and ECA was detected by incubation with horseradish peroxidase (HRP)-conjugated streptavidin (Vector Laboratories) for 30 min at RT. After three more washes with PBST, the optical density (OD) value at 450 nm was determined by treatment with tetramethylbenzidine substrate (TMB) for 15 min at RT, and the reactions were terminated using 2 N H_2_SO_4_. The samples were then read using an ELISA reader (Tecan, Kawasaki, Japan).

### 2.5. Hemagglutination Assay

The hemagglutination assay was performed using 50 µL of two-fold serially diluted virus (starting from 10^6^ PFU) in PBS with or without 10 µM oseltamivir carboxylate loaded onto a V-bottom 96-well plate. Then, 50 µL of 0.5% turkey red blood cells (RBCs) was added to the viruses, which were then incubated for 30 min at 4 °C and 37 °C for separate experimental sets. The final dilution in the wells that exhibited no agglutinated RBCs was used as the HA titer.

### 2.6. Virulence in Mice

Groups of BALB/c mice (female, 6 weeks old, n = 5) were anesthetized with isophorone and intranasally inoculated with 50 µL of 10^2^ or 10^3^ PFU of live viruses (RG pH1N1, RG pH1N1 (I365T/S366N), RG pH1N1 (I365E/S366D), or RG pH1N1 (I365T/S366N)), respectively. The body weight and survival rates of the mice were monitored for 14 days. Mice with a body weight loss of >25% were sacrificed according to the IACUC guidelines. The survival rates obtained from different infection doses were used to calculate the MLD50 values of the tested viruses.

### 2.7. Formalin-Inactivated Virus Preparation

Ten plates of MDCK cells (2 × 10^7^/plate) incubated in 20 mL of MEM-α with 0.5 µg/mL TPCK-trypsin were infected with viruses at MOI = 0.001. After 72 h of incubation at 37 °C, 200 mL of virus-containing culture supernatant was collected, centrifuged at 3000× *g* at 4 °C for 5 min to remove cell debris, and inactivated with 0.01% formalin (Sigma) at 4 °C for 24 h. The inactivated virus-containing solution was then concentrated to 30 mL using a 100 kDa spin column (Millipore, Burlington, MA, USA). Concentrated viruses were overlaid onto 5 mL of a 20% sucrose solution (w/v) dissolved in TNE buffer (10 mM Tris-HCl, 0.1 M NaCl, 1 mM EDTA, 10 mM, pH 7.4) in six ultracentrifugation tubes (Hitachi, Tokyo, Japan). After ultracentrifugation at 82,700× *g* and 4 °C for 2 h, the inactivated virus pellets were dissolved in 600 µL of PBS and stored at −80 °C [23,24]. The total protein concentration of the inactivated viruses was measured using the Bradford protein assay (Bio-Rad, Hercules, MA, USA).

### 2.8. Mouse Vaccination with Formalin-Inactivated Viruses

Groups of BALB/c mice (female, 6–8 weeks old, n = 5) were intramuscularly immunized with two doses of inactivated viruses containing 10 µg of total protein and 300 µg of alum adjuvant (Alhydrogel adjuvant; InvivoGen, San Diego, CA, USA) in a three-week interval. Antisera were collected 14 days after the second immunization and incubated at 56 °C for 30 min for complement inactivation. Antisera samples were stored at −20 °C before use.

### 2.9. NA Inhibition Assay

ELISA plates coated with 50 μg/mL (100 μL) of fetuin (Sigma) were incubated at 4 °C overnight. Then, the plates were washed three times with PBST and blocked with blocking buffer for 2 h. Viruses (pH1N1, H5N1, H3N2, or H7N9) were mixed with equal volumes of two-fold serially diluted serum samples for 1 h at 37 °C and transferred to ELISA plates coated with fetuin for 1 h at 37 °C. After washing three times with PBST, the desialylated O-linked glycans and desialylated N-linked glycans were probed with 100 μL of 2.5 μg/mL biotin-conjugated peanut agglutinin (PNA) (Vector Laboratories) and 100 μL of 1.25 μg/mL biotinylated lectin from erythrina cristagalli (ECA) (Vector Laboratories), respectively, in separate sets of experiments at RT for 1 h. After three washes with PBST, the binding of PNA and ECA was detected by incubation with horseradish peroxidase -conjugated streptavidin (Vector Laboratories) for 30 min at RT. After three more washes with PBST, the OD at 450 nm was determined by treatment with tetramethylbenzidine substrate (TMB) for 15 min at RT. The reactions were terminated using 2 N H_2_SO_4_ and the samples were read using an ELISA reader (Tecan). The serum dilutions that inhibited 50% of NA enzyme activity were defined as the IC50 values.

### 2.10. Virus-Neutralization Assay by Plaque Reduction

MDCK cells were seeded in DMEM with 5% FBS in 6-well plates for 48 h. Mouse sera were serially diluted two-fold in minimum essential medium-α (MEM-α) with 0.5 μg/mL TPCK-trypsin, and 30 μL of each diluted sample was co-incubated with 30 μL of pH1N1 virus (100 PFUs) at 37 °C for 1 h. The prepared MDCK cells were washed twice with PBS, and the medium was changed to 940 μL of MEM-α with 0.5 μg/mL TPCK-trypsin. The serum-virus mixture samples were added to the prepared cells and incubated at 37 °C for 1 h. The infected cells were washed with PBS and covered with 3 mL of overlay gel (0.5% low melting agarose in MEM-α with 0.5 µg/mL TPCK-trypsin) and incubated at 37 °C for 48 h for plaque formation. The cells were then fixed with 1 mL of 4% formalin (Sigma) for 6 h. After removing the overlay gel, the plaques were stained with 1% crystal violet. The percentage of virus neutralization in each well was calculated as follows: virus neutralization (%) = (number of viral plaques/number of viral plaques in the virus-only control groups) × 100%. The results were used to plot neutralization curves.

### 2.11. HI Assay

To eliminate the materials causing nonspecific erythrocyte aggregation, 10 µL of mouse serum was incubated with 30 µL of receptor-destroying enzyme (RDE; Denka Seiken) at 37 °C for 18 h. After another round of incubation at 56 °C for 30 min for RDE inactivation, 60 µL of PBS was added to the serum–enzyme mixture. The serum–enzyme mixtures were serially diluted two-fold in 25 µL of PBS in V-bottomed 96-well plates and incubated with 4 HA units of pH1N1 viruses in 25 µL of PBS at RT for 30 min. Then, 50 µL of 0.5% turkey red blood cells was added. The mixture was incubated at 4 °C for 30 min, and the HI titers were determined as the final dilution that inhibited hemagglutination.

### 2.12. Statistics

Statistical analyses were performed using GraphPad Prism (GraphPad Software, Inc., San Diego, CA, USA). The statistical significance of differences between the groups was assessed using one-way analysis of variance (ANOVA) with Tukey’s or Holm–Sidak multiple comparison tests. Differences with a *p*-value of less than 0.05 (*), 0.01 (**), 0.001 (***), and 0.0001 (****) were considered statistically significant.

## 3. Results

### 3.1. Analysis of the NA 370-Loop Amino Acid Sequences of H1N1 Viruses

**Figure 1:**
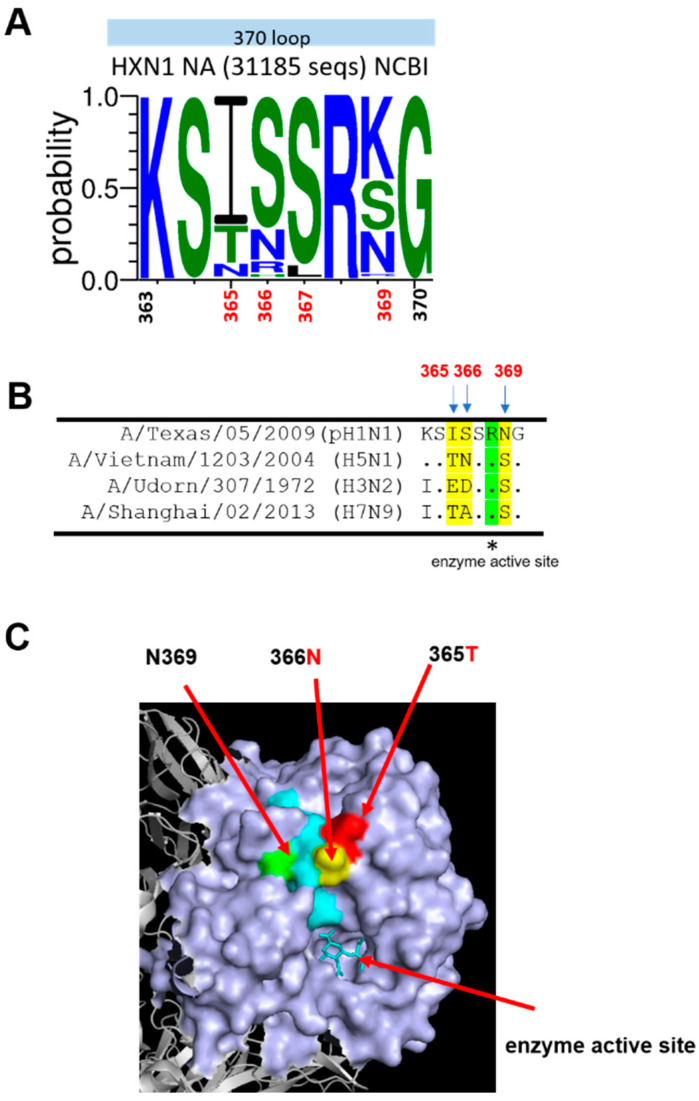
NA 370-loop amino acid sequence analysis. (**A**) WebLogo of 370-loop was generated based on 31,185 sequences of N1NA from HXN1 viruses from NCBI using the WebLogo3 website. (**B**) Amino acid sequence alignment of NA sequences from A/Texas/05/2009 (H1N1), A/Vietnam/1203/2004 (H5N1), A/Udorn/307/1972 (H3N2), and A/Shanghai/02/2013 (H7N9). (**C**) 3D structure of a monomer of N1NA of pH1N1 (PDB: 4b7r) The 370-loop is shown in cyan. The 365, 366, and 369 residues are shown in red, yellow, and green, respectively.

The amino acid sequences of the NA 370-loop at residues 363–370 from 31,185 HXN1 virus strains were analyzed using WebLogo 3 (http://weblogo.threeplusone.com/create.cgi) accessed on 16 May 2022. The analysis was used to calculate the percentage of amino acid residues at 365 for I (70%), T (20%), and N (10%); at 366 for S (72%), N (16%), R (8%), and H (4%); at 367 for S (92%) and L (8%); at 369 for K (44%), S (26%), N (22%), and R (4%) (Figure 1A). We previously demonstrated that immunization with recombinant NA (pH1N1, A/Texas/05/2009) proteins with I365T/S366N mutations elicited cross-reactive NA-inhibition antibodies against the homologous pH1N1 and three heterologous H3N2, H5N1, and H7N9 viruses [12]. The NA 370-loop amino acid sequences of pH1N1 (A/Texas/05/2009), H5N1 (A/Vietnam/1203/2004), H3N2 (A/Udorn/307/1972), and H7N9 (A/Shanghai/02/2013) are listed in Figure 1B. As shown in the 3D structure of the NA protein (PDB:4b7r) [6], the I365T/S366N mutations from pH1N1 (A/Texas/05/2009) switched to H5N1(A/Vietnam/1203/2004) at the NA 370-loop are located at the upper surface near the enzyme activity site (Figure 1C).

### 3.2. Construction of RG pH1N1 Viruses with the NA 370-Loop Mutations

**Figure 2:**
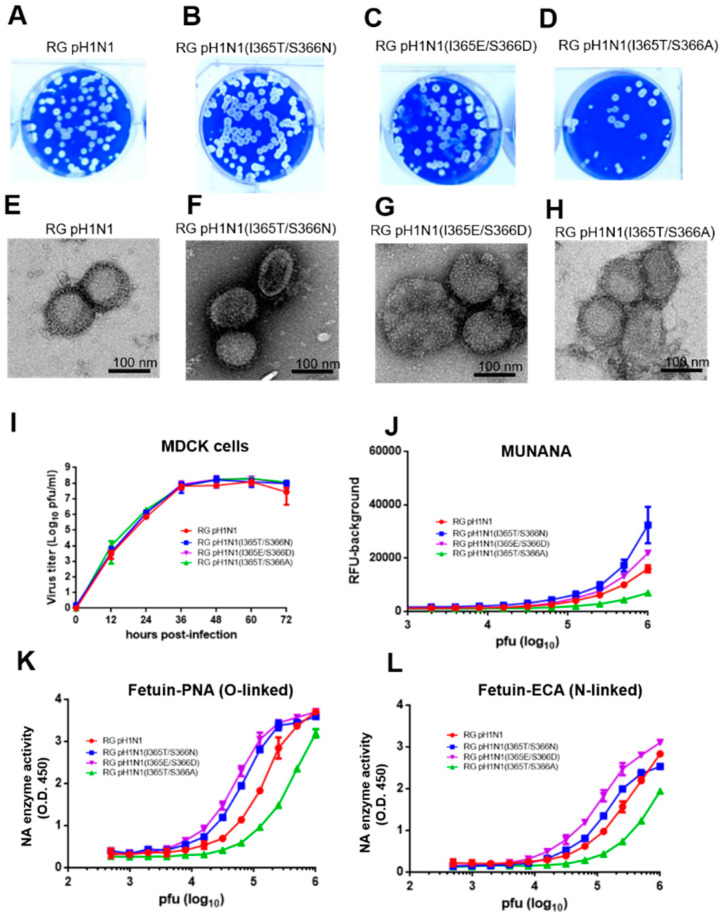
Generation and characterization of pH1N1 RG viruses. Viral plaques of (**A**) RG pH1N1, (**B**) RG pH1N1(I365T/S366N), (**C**) RG pH1N1(I365E/S366D), and (**D**) RG pH1N1(I365T/S366A). TEM images of (**E**) RG pH1N1, (**F**) RG pH1N1(I365T/S366N), (**G**) RG pH1N1(I365E/S366D), and (**H**) RG pH1N1(I365T/S366A). (**I**) Viral growth curves in MDCK cells infected at MOI = 0.1 at different hours post-infection. (**J**) Viral NA enzyme activity on monovalent MUNANA substrates. (**K**) Viral NA enzyme activities on O-linked SIA of multivalent fetuin substrate. (**L**) Viral NA enzyme activities on the N-linked SIA of multivalent fetuin substrate.

The PR8 eight plasmids-based RG system was used to generate the wild-type pH1N1 RG virus and three NA 370-loop mutant viruses: RG pH1N1(I365T/S366N), RG pH1N1(I365T/S366A), and RG pH1N1(I365E/S366D). All of these RG viruses were rescued (Figure 2A–D). The morphology of these four RG viruses was roughly spherical and pleomorphic with a spike signature by TEM visualization (Figure 2E–H). The growth kinetics of these four RG viruses in MDCK cells were similar as shown by the virus titer measured at different hours post-infection (Figure 2I).

### 3.3. NA Activity for RG pH1N1 Viruses with the NA 370-Loop Mutations

The NA enzyme activity of these four pH1N1 RG viruses was measured using the monovalent substrate MUNANA or the multivalent substrate fetuin [12,22,23]. The MUNANA assay indicated that both the RG pH1N1 (I365T/S366N) and RG pH1N1 (I365E/3S66D) viruses had a higher NA activity, but the RG pH1N1 (I365T/S366A) virus had a lower NA activity than the wild-type RG pH1N1 virus (Figure 2J). The O-linked fetuin-PNA and N-linked fetuin-ECA assays also showed the order of NA enzyme activity following RG pH1N1(I365E/S366D) > RG pH1N1(I365T/S366N) > RG pH1N1 > RG pH1N1(I365T/S366A) (Figure 2K,L). The differences in the NA enzyme activity of these mutant viruses measured using multivalent substrates (O-linked or N-linked) were more significant than the values measured using the monovalent substrate MUNANA. The results demonstrated that the I365T/S366N and I365E/S366D NA mutant RG viruses had an increased viral NA enzyme activity, but the I365T/S366A RG mutant RG virus had a reduced NA enzyme activity.

### 3.4. NA Kinetic Parameters for RG pH1N1 Viruses with the NA 370-Loop Mutations

**Figure 3:**
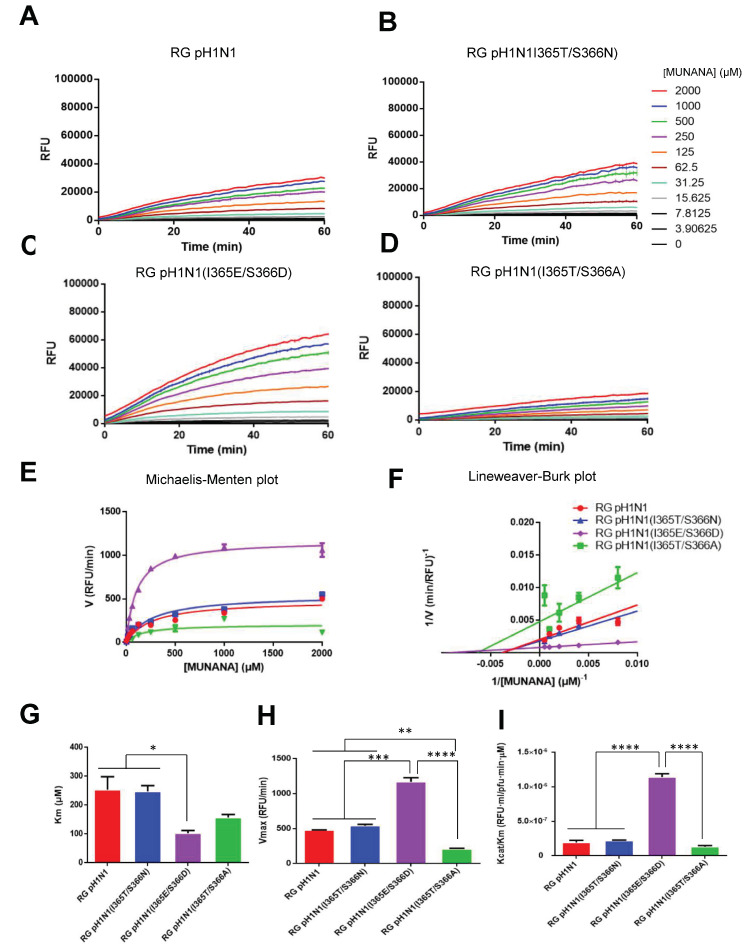
Viral NA enzyme kinetics. To determine the viral NA enzyme activities in real-time, the emission signals from different concentrations of MUNANA cleaved by NA of (**A**) RG pH1N1, (**B**) RG pH1N1(I365T/S366N), (**C**) RG pH1N1(I365E/S366D), and (**D**) RG pH1N1(I365T/S366A) were measured every 60 s for 1 h. (**E**) Michaelis–Menten plots of viral NA enzyme activities. (**F**) Lineweaver–Burk plots of viral NA enzyme activity. (**G**) Vmax, (**H**) Km, and (**I**) Kcat/Km of each viral NA calculated using Lineweaver–Burk plots. Statistical tests for multiple comparisons in (**G**–**I**) were analyzed using one-way ANOVA with Tukey’s or Holm–Sidak’s multiple comparison tests. (* *p* < 0.05, ** *p* < 0.01, *** *p* < 0.001 and **** *p* < 0.0001). Error bars are plotted as standard deviation from the mean value.

The enzyme kinetic parameters of these four RG viruses were further measured using the MUNANA assay. Time course data for each concentration of the MUNANA substrate were recorded for the RG pH1N1, RG pH1N1(I365T/S366N), RG pH1N1(I365T/S366A), and RG pH1N1(I365E/S366D) viruses (Figure 3A–D). The velocities of the NA enzymes of these RG viruses were plotted using a Michaelis–Menten plot (Figure 3E). By plotting 1/V0 (reciprocal of initial velocity) against 1/S0 (reciprocal of substrate concentration), the Lineweaver–Burk plot was obtained (Figure 3F). The kinetic parameters Michaelis–Menten constant (Km) and maximum velocity of substrate conversion (Vmax) were calculated from the Lineweaver–Burk plot. The results showed that pH1N1 (I365E/S366D) and pH1N1 (I365T/S366A) viruses had reduced Km values for enzyme affinity constant compared to the RG pH1N1 virus (Figure 3G). However, the RG pH1N1(I365E/S366D) virus had higher Vmax values than the RG pH1N1 and RG pH1N1(I365T/S366N) viruses, whereas RG pH1N1(I365T/S366A) had a lower Vmax value than that of the RG pH1N1 virus (Figure 3H). In addition, the RG pH1N1(I365E/S366D) virus had a higher Kcat/Km value than the RG pH1N1, RG pH1N1(I365T/S366N), and RG pH1N1(I365T/S366A) viruses.

### 3.5. HA Titers for RG pH1N1 Viruses with the NA 370-Loop Mutations

**Figure 4:**
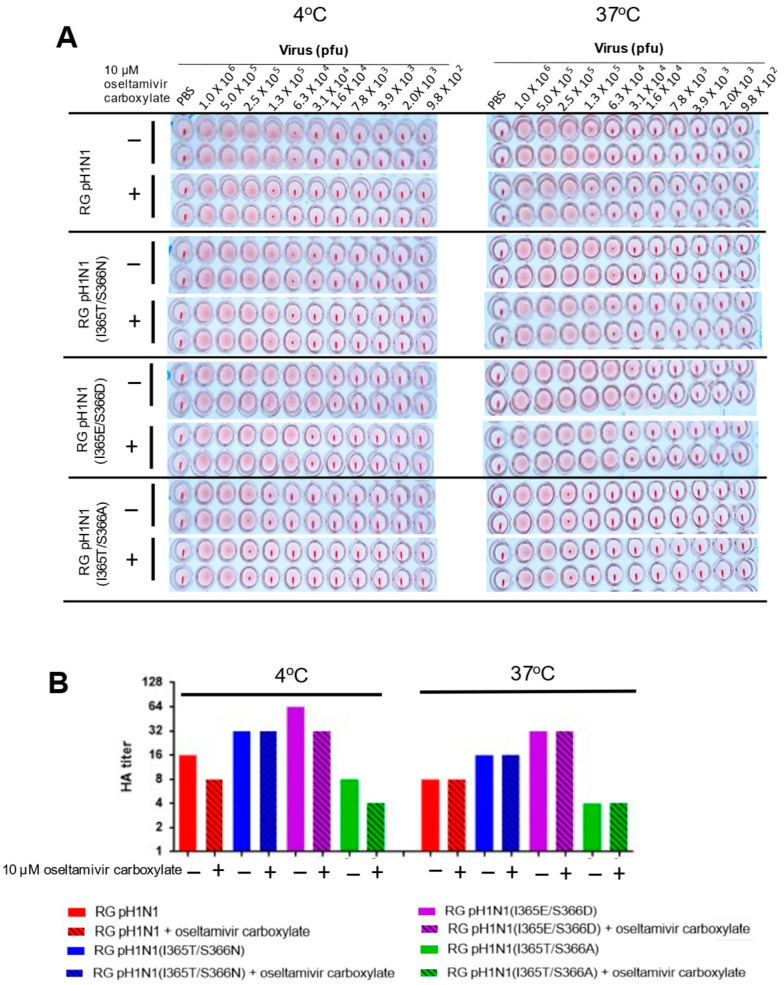
HA titers of RG pH1N1, RG pH1N1 (I365T/S366N), RG pH1N1 (I365E/S366D, and RG pH1N1 (I365T/S366A). (**A**) HA titers of RG pH1N1, RG pH1N1 (I365T/S366N), RG pH1N1 (I365E/S366D) and RG pH1N1 (I365T/S366A) with or without oseltamivir carboxylate treatment determined by hemagglutination assays using 0.5% turkey RBCs at 4 °C and 37 °C. (**B**) The HA titers plotted as a bar chart.

Since the NA activity of influenza viruses can affect the HA–NA balance, which in turn affects the initiation of viral infection, viral fitness, and cross-species transmission [24], we measured the HA titers of these RG pH1N1 viruses using turkey RBCs in the presence or absence of 10 µM oseltamivir carboxylate at 4 °C or 37 °C. The results showed that under all of the conditions tested, the RG pH1N1(I365T/S366N) and RG pH1N1(I365E/S366D) viruses had higher HA titers than the RG pH1N1 virus although these values are not statistically significant, while the RG pH1N1(I365T/S366A) virus had the lowest titers (Figure 4A,B). The increase or decrease in HA titers was found to roughly correlate with the increase or decrease in the measured NA activity for these NA 370-loop mutant viruses.

### 3.6. Mouse Virulence for RG pH1N1 Viruses with the NA 370-Loop Mutations

**Figure 5:**
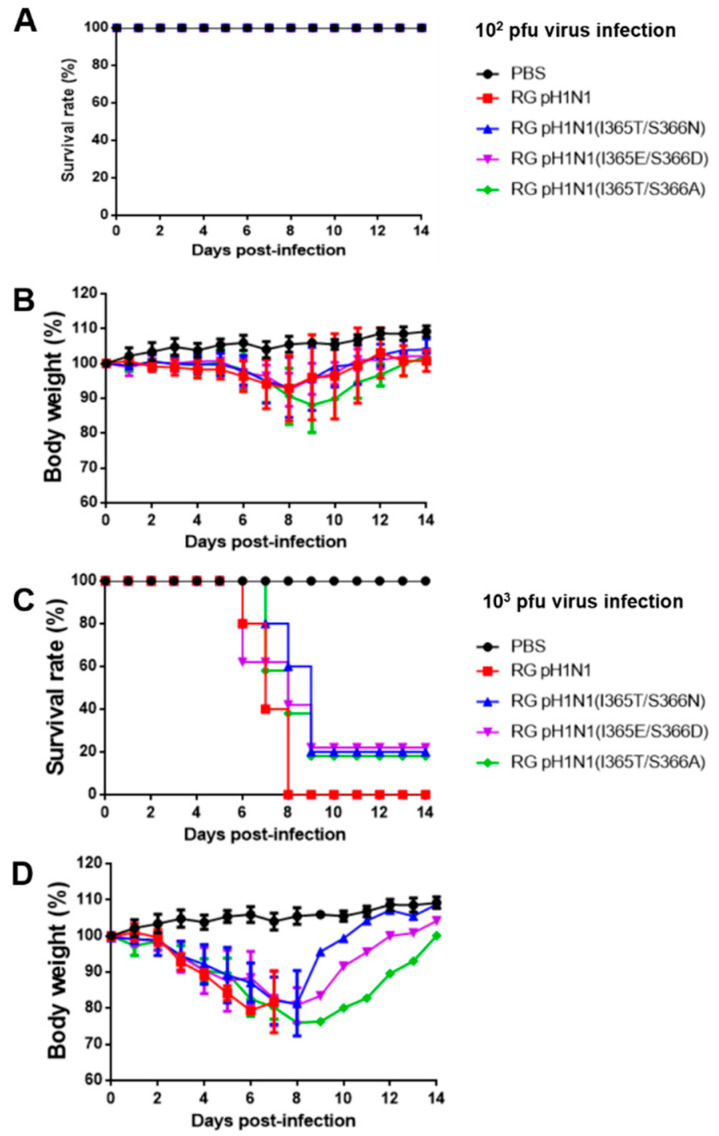
Virulence of the RG pH1N1, RG pH1N1(I365T/S366N), RG pH1N1 (I365E/S366D), and RG pH1N1(I365T/S366A) viruses. Five mice from each group were intranasally infected with RG pH1N1, RG pH1N1(I365T/S366N), RG pH1N1(I365E/S366D), or RG pH1N1(I365T/S366A). (**A**) Survival rates of mice inoculated with 10^2^ viruses. (**B**) Body weight of mice inoculated with 10^2^ viruses. (**C**) Survival rates of mice infected with 10^3^ viruses. (**D**) Body weight of mice infected with 10^3^ viruses.

To determine whether the increased NA and HA titers observed in these RG pH1N1 viruses may also affect their virulence, 10 groups of BALB/c mice (n = 5 per group) were intranasally inoculated with 10^2^ and 10^3^ pfu RG viruses alone with the PBS control, and their survival rates and body weight recovery were recorded for 14 days. For 10^2^ pfu inoculation, the results indicated that a 100% survival rate was found for all the investigated groups (Figure 5A), while the loss of body weight and its recovery for 14 days was more significant for the RG pH1N1(I365T/S366A) group than for the RG pH1N1, RG pH1N1(I365T/S366N), and RG pH1N1(I365E/S366D) groups (Figure 5B). For 10^3^ pfu inoculation, the results showed that the RG pH1N1(I365T/S366N), RG pH1N1(I365T/S366A), and RG pH1N1(I365E/S366D) groups had a survival rate of 20% compared to 0% for the RG pH1N1 and 100% for the PBS control (Figure 5C). The body weight loss was recovered for three NA 370-loop mutants but not the wild-type RG pH1N1 (Figure 5D). Therefore, the RG pH1N1, RG pH1N1(I365T/S366N), RG pH1N1(I365T/S366A), and RG pH1N1(I365E/S366D) viruses were less virulent than the wild-type RG pH1N1 virus.

### 3.7. NA-Inhibition, HI, and Virus-Neutralizing Antibodies Elicited by Inactivated RG pH1N1 Viruses with the NA 370-Loop Mutations

**Figure 6:**
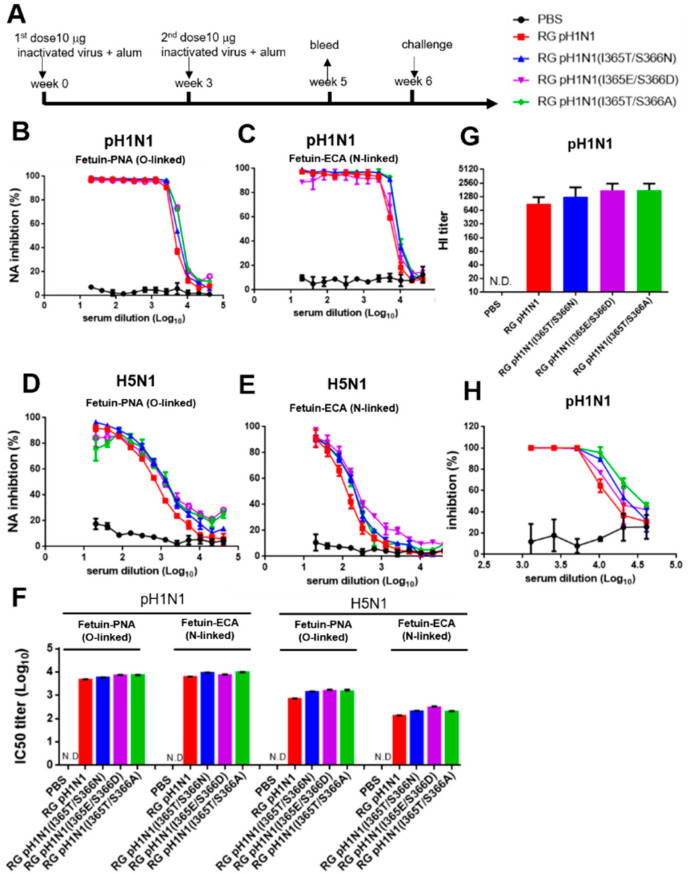
NA-inhibition, HA-inhibition, and virus-neutralizing antibodies elicited by the inactivated RG pH1N1 viruses with NA 370-loop mutations. (**A**) Groups of BALB/c mice (n = 5 per group) were intramuscularly immunized with each group of inactivated viruses containing 10 µg total protein plus alum adjuvant using a two-dose regimen. Antisera were collected on week 5. NA-inhibition antibody curves against (**B**) pH1N1 (NA activity on O-linked SIA), (**C**) pH1N1 (NA activity on N-linked SIA), (**D**) H5N1 (NA activity on O-linked SIA), and (**E**) H5N1 (NA activity on N-linked SIA). (**F**) IC50 titers of NA-inhibition antibodies against pH1N1 and H5N1 viruses. (**G**) HI titers against pH1N1 virus. (**H**) Virus-neutralization inhibition for pH1N1 virus.

The culture supernatants were collected from MDCK cells infected with each RG pH1N1 virus and then treated with 0.01% formalin for 24 h, concentrated, and purified by 20% sucrose ultracentrifugation to obtain the inactivated viruses for immunization. Groups of BALB/c mice (n = 5 per group) were intramuscularly immunized with each group of inactivated viruses containing 10 µg total protein plus alum adjuvant using a two-dose regimen (Figure 6A). Antisera were collected two weeks after the second-dose immunization to determine the titers of NA-inhibition, HI, and virus-neutralizing antibodies. The antisera from the four inactivated virus groups, but not the PBS control, showed dose-dependent NA inhibition against the homologous pH1N1 (Figure 6B,C) and the heterologous H5N1 viruses (Figure 6D,E). The corresponding IC50 titers of the NA-inhibition antibodies were 3.7–3.9 log10 against the pH1N1 virus and 2.8–3.2 log10 (O-linked) and 2.1–2.5 log10 (N-linked) against the H5N1 virus (Figure 6F). No significant differences in NA-inhibition antibody titers were found between the inactivated RG pH1N1 and inactivated RG pH1N1 NA 370-loop mutant viruses. Furthermore, no detectable levels of NA-inhibition antibodies against the heterosubtypic H3N2 and H7N9 viruses were observed (data not shown). We also examined the HA-inhibition titers of these antisera using turkey RBCs, which showed a gradual increase in the immunized groups of RG pH1N1, RG pH1N1(I365T/S366N), RG pH1N1(I365E/S366D), and RG pH1N1(I365T/S366A) (Figure 6G). The virus-neutralizing antibody titers were also determined using a plaque assay, showing similar results, with a slight increase in the dose-dependent inhibition by RG pH1N1, RG pH1N1(I365T/S366N), RG pH1N1(I365E/S366D), and RG pH1N1(I365T/S366A) (Figure 6H). No cross-reactive HA-inhibition titers were detected in these antisera against the H5N1, H3N2, and H7N9 viruses (data not shown). Overall, the inactivated viruses of RG pH1N1(I365T/S366N), RG pH1N1(I365E/S366D), and RG pH1N1(I365T/S366A) compared to RG pH1N1 were found to elicit similar titers of NA-inhibition antibodies against pH1N1 and H5N1 viruses, but not H3N2 and H7N9 viruses as well as of HI and virus-neutralizing antibody titers against the pH1N1 viruses.

## 4. Discussion

HA and NA are two major antigens of the influenza A virus. Although both HA and NA undergo antigenic drift, NA experiences less antigenic change than HA [25]. Although NA-inhibition antibodies were shown to provide cross-protection against heterologous viruses, this effect was restricted in viruses carrying the same influenza NA subtype [18]. For instance, the H3N2 DNA vaccine was reported to protect against heterologous H3N2 viruses, but not against the H1N1 virus [26]. The key amino acids of the cross-reactive epitope (s) against H1N1 and H5N1 viruses were located at the seven upper loops surrounding the enzyme activity site (14). We previously demonstrated that recombinant NA protein with I365T/S366N mutation elicited cross-reactive NA-inhibition antibodies against the homologous pH1N1, the heterologous H5N1, and the heterosubtypic H3N2, and H7N9 viruses [12]. In this study, we used RG technology to engineer the wild-type pH1N1 (RG pH1N1) and three NA 370-loop mutants (I365T/S366N, I365E/S366D, and I365T/S366A) and investigated their NA enzyme activity, HA titers, mouse virulence, and inactivated-virus immunogenicity.

Our studies demonstrated that the I365T/S366N and I365E/S366D NA mutant viruses had an increased viral NA enzyme activity, but the I365T/S366A RG mutant virus had a reduced NA enzyme activity, based on the cleavage of SIA on MUNANA and the cleavages of O- and N-linked SIA on fetuin as a multivalent substrate. These three 370-loop mutant viruses (I365T/S366N, I365E/S366D, and I365T/S366A) did not show differences in their viral growth kinetics in MDCK cells (Figure 2I), and the sensitivity to oseltamivir had no difference for HA titers (Figure 4B). These results are different from other reports to show that mutant H1N1 and H3N2 viruses with reduced NA enzyme activity had decreased viral growth kinetics, reduced oseltamivir sensitivity, and lowered virulence, including the H274Y NA mutation of A/Texas/36/91 (H1N1) [26], the H275Y NA mutation of A/England/195/09 (H1N1) [27], the I427T NA mutation of A pH1N1 [28], and the E119V and R292K NA mutations of A/Wuhan/359/95 (H3N2) [29]. Furthermore, the three mutant RG viruses (I365T/S366N, I365E/S366D, and I365T/S366A) were also found to display an increase or decrease in HA titers that was roughly correlated with an increase or decrease in NA activity in these NA 370-loop mutant viruses, suggesting the possibly altered the amounts of HA and NA on the virion’s surface or the HA–NA receptor balance for viral fitness [2,30]. It was reported that the influenza virus with enhancing NA enzyme activity can restore the replication by low-affinity receptor binding HA mutant viruses [31]. The altered HA–NA receptor balance for influenza A viruses can be also affected by virion lengths and the HA/NA ratios due to the low-fidelity assembly [32]. Therefore, further studies are needed to determine whether the various levels of NA enzyme activities and HA titers of these three mutant viruses (I365T/S366N, I365E/S366D, and I365T/S366A) are related to virus assembly.

Our findings of NA-inhibition antibodies elicited by the inactivated I365T/S366N mutant virus were not consistent with our previous study using mutant I365T/S366N NA protein for immunization [12]. Immunization with the inactivated viruses of RG pH1N1, RG pH1N1(I365T/S366N), RG pH1N1(I365T/S366A), and RG pH1N1(I365E/S366D) was found to elicit similar titers of NA-inhibition antibodies against pH1N1 and H5N1 viruses, respectively (Figure 6B–E). No detectable NA-inhibition antibodies against the heterosubtypic H3N2 and H7N9 viruses were found (data not shown). It was reported that formalin-inactivated influenza virus contained approximately 0.62% NA content compared to 25.58% HA content in a single virion surface (i.e., 645 μg inactivated virus containing 165 μg HA and 4 μg NA) [33]. Based on this estimation, one dose of 10 μg inactivated pH1N1 RG virus in the immunization study was composed of approximately 2.558 μg HA and 0.062 μg NA for inactivated virus immunization. The results may be due to the insufficient NA amounts for presenting the cross-reactive epitope(s) using inactivated virus immunization as compared to NA protein immunization. Therefore, future studies using NA-based virus-like particles or some other way to increase the NA presentation can provide useful information for NA-based influenza vaccine development.

## 5. Conclusions

In conclusion, this study showed that the RG viruses carrying the NA 370-loop mutations, including I365T/S366N, I365E/366D, and I365T/S366A affected viral NA enzyme activity and virulence in mice, but had no effect on virus growth kinetics in MDCK cells or oseltamivir sensitivity. Immunization with formalin-inactivated viruses of these NA 370-loop mutants compared to the wild-type RG pH1N1 virus elicited similar titers of NA-inhibition antibodies against pH1N1 and H5N1 viruses. Future studies using NA-based virus-like particles or some other way to increase the NA presentation could provide useful information for NA-based influenza vaccine development.

## Data Availability

The raw data supporting the conclusions of this article will be made available by the authors, without undue reservation.
